# Syndromic Surveillance for Influenzalike Illness in Ambulatory Care Setting

**DOI:** 10.3201/eid1010.030789

**Published:** 2004-10

**Authors:** Benjamin Miller, Heidi Kassenborg, William Dunsmuir, Jayne Griffith, Mansour Hadidi, James D. Nordin, Richard Danila

**Affiliations:** * Minnesota Department of Health, Minneapolis, Minnesota, USA;; † Minnesota Department of Agriculture, St. Paul, Minnesota, USA;; ‡ University of New South Wales, Sydney, Australia;; § HealthPartners Research Foundation, Minneapolis, Minnesota, USA

**Keywords:** Bioterrorism, Surveillance, ICD-9, Syndrome, Research

## Abstract

Conventional disease surveillance mechanisms that rely on passive reporting may be too slow and insensitive to rapidly detect a large-scale infectious disease outbreak; the reporting time from a patient's initial symptoms to specific disease diagnosis takes days to weeks. To meet this need, new surveillance methods are being developed. Referred to as nontraditional or syndromic surveillance, these new systems typically rely on prediagnostic data to rapidly detect infectious disease outbreaks, such as those caused by bioterrorism. Using data from a large health maintenance organization, we discuss the development, implementation, and evaluation of a time-series syndromic surveillance detection algorithm for influenzalike illness in Minnesota.

## Abstract 2

Detection algorithm using proxy data for a bioterrorism agent release and historical data for influenza was effective.

Rapid identification of a bioterrorism-related outbreak poses challenges to traditional public health disease surveillance (*1*). At the individual level, the nonspecific prodrome of many diseases caused by bioterrorism agents requires the disease to be recognized by an astute clinician (*2**,**3*). At the population level, an intentional release of a bioterrorism agent may require that disease clusters of syndromes, such as influenzalike illnesses (ILI), be recognized through recently developed nontraditional surveillance mechanisms, such as syndromic surveillance (*4**–**7*). For instance, increases in ILI shown by syndromic surveillance could indicate undiagnosed inhalation anthrax or pneumonic plague.

Various data sources can be used to construct syndromic surveillance systems. Existing patient data sources, such as emergency room chief complaints, ambulance dispatch data, and clinical diagnosis data, have been used (*8**–**12*). Metadata collection systems that incorporate emergency room syndromes, private practice billing codes grouped into syndromes, and veterinary syndromes also exist (*7*). Other existing data sources that are potentially suitable for syndromic surveillance include calls to poison control centers, over-the-counter and prescription medication sales (*7**,**13*), nurse help-line telephone logs (*7**,**14*), and absenteeism in schools (*7*). In this study, we evaluate the use of data from an ambulatory care clinic network to detect increases of ILI using a time-series autoregressive and cumulative sum (CUSUM)–based detection algorithm.

## Methods

### Data Source

Data in this study are from the HealthPartners Medical Group (HPMG), which is a family of nonprofit Minnesota healthcare organizations that serves approximately 240,000 patients in the Minneapolis-St. Paul Metropolitan Area. HPMG is a current partner in the National Bioterrorism Syndromic Surveillance Demonstration Program (*12*).

Investments in technology infrastructure allow HPMG to digitally record International Classification of Diseases, Revision 9 (ICD-9) data from patient visits to network clinics within approximately 24 hours of a patient's initial visit. The need to develop validated and standardized methods for syndromic surveillance is a current challenge to the field. A framework for evaluating syndromic surveillance systems has been developed, which provides a general approach to comparing and contrasting aspects of syndromic surveillance systems (*15*). We have adapted some of these recommendations and have identified the following six criteria that should be satisfied before developing syndromic surveillance detection algorithms: 1) data are collected and should exist for reasons other than bioterrorism surveillance; 2) data should be recorded and accessible in a recognized, consistent, and electronic format; 3) data should be available for analysis shortly after the patient's initial visit; 4) sufficient historical data sources should be available that represent a reasonably static and definable population; 5) syndromes should be validated against existing traditional data sources; and 6) thresholds set for these systems should achieve high sensitivity and positive predictive value. Based on the framework for evaluating syndromic surveillance systems, we assess the appropriateness of ambulatory-care encounter data from HPMG.

To satisfy the first criterion, these data are collected at HPMG shortly after the patient's initial visit; then identification is removed and the data transferred to the Minnesota Department of Health for analysis without additional work for physicians, clinic staff, or patients. For each scheduled, same-day, or urgent care patient encounter, ICD-9 codes are collected, recorded, and stored in a standardized electronic format, which fulfills the second criterion. Multiple ICD-9 codes may be recorded for each patient (e.g., 786.2 cough and 780.6 fever). The ICD-9 format consistency allows for reliable syndrome classification and analysis. Other possible sources of data that have been reliably classified into syndrome categories and could be used in this type of time-series analysis include chief complaint text fields and Health Level 7 (HL-7) messaging data (*16*). ICD-9 encounter data and nonidentifying demographic information are queried daily from the HPMG central patient database and sent to the Minnesota Department of Health through secure file transfer protocol. Fulfilling the third criterion, an advanced electronic patient-tracking system allows for >90% of the encounter data at HPMG to be available in HPMG databases within 24 hours of the clinic encounter. In addition to the daily transmitted data, historical encounter data beginning in April 1999 are used in the analysis. These historical data represent a consistently insured population within the clinic network with minimal immigration into or emigration from the HPMG network, thus satisfying the fourth criterion for this type of autoregressive time-series analysis. Had HPMG experienced substantial changes in its insured population, the underlying statistical assumptions necessary for this analysis would have been violated (*17*). Validation against a traditional data source, the fifth criterion, and the selection of an appropriate threshold, the sixth criterion, will be discussed below in the Validation section.

### Models and Analysis

We used an autoregressive model (PROC AUTOREG) to model the square root of the daily counts of ILI to the HPMG clinics in a 3-year historical period (*18*). Sample SAS code with tests for autocorrelation and stepwise autoregression is provided in the Appendix. The model closely resembles ordinary linear regression, but instead of the usual regression model, the following autoregressive error model is used:


_
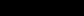
_



_
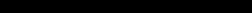
_



_
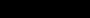
_


The notation_
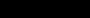
_ indicates that each ε*_t_* is normally and independently distributed with mean 0 and variance *σ*^2^. By simultaneously estimating the regression coefficients *β* and the autoregressive error parameters *φ_i_*, the model corrects the regression estimates for autocorrelation, a common problem in time-series data.

In the model, we include an indicator for weekend or weekday, an indicator of the day as a regular or national holiday, and indicators for a sine and cosine function for seasonal adjustment. The model also includes a seventh-order autoregressive error model, selected by stepwise regression. In each case, the terms contribute significantly to the fit of the model (p < 0.05).

The predicted residuals from this model are then analyzed by using the cumulative sums method (PROC CUSUM) (*18**,**19*). Initially used in the manufacturing industry, CUSUM has been used for *Salmonella* surveillance in the United States and for influenza surveillance in the United Kingdom (*20**–**22*). The method has properties making it well suited for disease outbreak detection. It can quickly detect small shifts from the process mean, provide estimates of when the change occurred, and estimate the magnitude of change (*17**,**23**–**25*).

### Validation

Because syndromic surveillance attempts to identify disease outbreaks before a definitive diagnosis is made, assessing the validity of the ILI syndrome is difficult. The actual cause of many signals generated by this system may never be known because many patients are never requested to submit specimens for laboratory testing.

We assessed the validity of the HPMG ILI syndrome category by comparing ILI visits in the HPMG network to deaths from pneumonia and influenza in the core seven-county Minneapolis-St. Paul metropolitan area over the same time period. ICD-9 codes that describe ILI were selected (Table). We also associated increases in ILI with the known onset of influenza season by comparing influenza isolates and hospital laboratory data.

**Table:** Influenzalike illness ICD-9 codes selected for analysis^a^

ICD-9 code	Description	ICD-9 code	Description
079.99	Viral infection	490	Bronchitis
307.81	Headache, tension	493.00	Asthma/ROAD
372.30	Conjunctivitis	493.90	Asthma
460	Rhinitis, acute	780.4	Dizziness
461.1	Sinusitis, acute, NOS	780.6	Fever
462	Pharyngitis	780.79	Fatigue/weakness/malaise
464.0	Laryngitis	782.5	Cyanosis
464.4	Croup	784.0	Headache
465.9	URI	785.6	Lymphandenopathy
466.19	Bronchitis, acute	786.07	Wheezing
472.0	Rhinitis	786.09	Dyspnea
477.9	Rhinitis, allergic	786.2	Cough
483.0	Pneumonia, mycoplasm	786.50	Pain, chest
486	Pneumonia	787.02	Nausea
496	COPD	787.03	Vomiting
487.1	Influenza		

^a^ROAD, reversible obstructive airways disease; NOS, not otherwise specified; URI, upper respiratory tract infection; COPD, chronic obstructive pulmonary disease.

### System Testing

Data pertaining to the incubation period for inhalational exposure to most potential bioterrorism agents are limited. Therefore, evaluation of this system used hypothetical scenarios in which additional ICD-9 counts were added to existing clinical data to determine the number of excess cases necessary to trigger a signal. In an approach adapted from Goldenberg, our hypothetical scenario uses data gathered from the only documented large-scale aerosol release of weapons-grade *Bacillus anthracis* spores (*13*).

In April and May 1979, an unusual outbreak of inhalational anthrax occurred in the city of Sverdlovsk in the former Union of Soviet Socialist Republics. The outbreak was originally ascribed to consuming contaminated meat, but investigation by Soviet and international scientists subsequently linked the outbreak to an accidental aerosolized release of anthrax spores from a nearby military facility (*26*). The inadvertent release, which may have contained as little as several milligrams of spores, caused 77 confirmed inhalational anthrax cases and 68 deaths in 43 days.

To test our model, we constructed three hypothetical scenarios on the basis of data available from the Sverdlovsk release. We made several assumptions in constructing these scenarios. First, a point-source release pattern similar to that observed in Sverdlovsk occurs in the downtown area of the Minneapolis-St. Paul metropolitan area during a weekday when most of the population is at work. This scenario will effectively disperse exposed persons to most clinics in the HPMG network, if one assumes that exposed persons will seek care at a clinic near their residence. Second, a subset of those exposed to the release will visit an HPMG clinic on the date of symptom onset and in a manner identical to those exposed in the Sverdlovsk release. This assumption effectively replicates the incubation periods experienced by those in the Sverdlovsk release and adds these additional cases to the daily totals of ILI observed in HPMG clinics. The third assumption increases the overall numbers of patients seeking care in the HPMG network from this exposure to 308 during a 43-day period, four times the number of confirmed ill in the Sverdlovsk release. The anthrax release and the resulting increase in ILI were modeled for three different time periods to determine the effect of season, day of the week, holidays, and naturally occurring ILI on the ability to detect the outbreak.

## Results

### Validation

**Figure 1:**
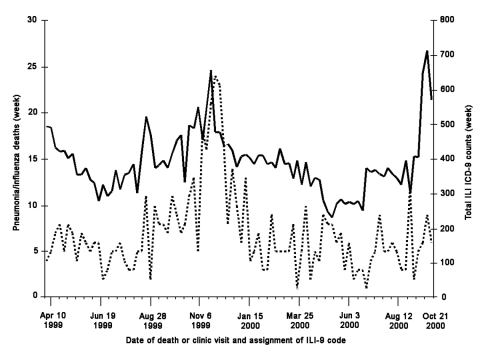
Weekly totals of HealthPartners Medical Group influenzalike illness ICD-9 counts (solid line) and Minneapolis-St. Paul metropolitan area weekly influenza and pneumonia deaths (broken line) April 10, 1999, through December 29, 2000.

The seasonal variation in HPMG ICD-9 ILI counts is similar to the variation in deaths from pneumonia and influenza in the core seven-county Minneapolis-St. Paul metropolitan area, as reported by the Minnesota Department of Health (Figure 1). To satisfy the fifth criterion for establishing syndromic surveillance, i.e., validating syndromes against existing traditional data sources, death data from April 10, 1999, to December 29, 2000, were compared to ILI ICD-9 counts over the same period. Visual comparison of these data in Figure 1 suggests that ICD-9 ILI counts rose several weeks before the peak in deaths. ILI syndrome validity was determined to be acceptable, as Pearson correlation results were significant between weekly influenza and pneumonia deaths and ILI clinical encounters in the same week (0.41) and the previous week (0.41).

**Figure 2:**
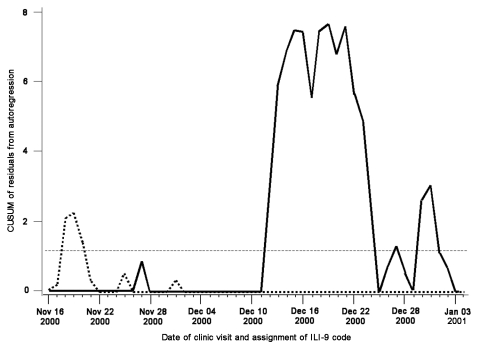
Cumulative sum (CUSUM) chart signaling a significant signal corresponding to a confirmed influenza A outbreak occurring December 2000 and January 2001. CUSUM decision interval (horizontal broken line); CUSUM chart signals 24 days earlier when the analysis is stratified by age: >65 years (dotted line) and all ages (solid line).

Formal calculations of sensitivity and positive predictive value were not conducted in this study. Calculation of the appropriate threshold used in the detection algorithm was determined qualitatively by adjusting the model parameters to detect the onset of influenza season. Figure 2 illustrates a large and continuous signal that was retrospectively observed beginning on December 12, 2000, and continuing until December 25, 2000. This alarm corresponds to a large ILI outbreak in the Minneapolis-St. Paul metropolitan area and was possibly associated with increased influenza A and respiratory syncytial virus infection. A hospital in the HPMG network reported above average submission and testing of isolates corresponding to these organisms during December 2000 and January 2001.

### System Testing

**Figure 3:**
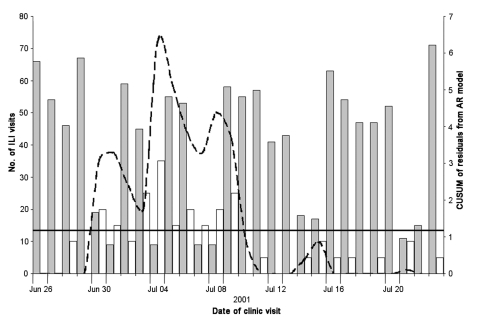
Cumulative sum (CUSUM) control chart of a hypothetical anthrax release occurring June 26, 2001. CUSUM of the residuals (broken line) is charted over the observed number of influenzalike (ILI) visits to the HealthPartners Medical Group (gray bars) and the additional outbreak-associated ILI cases (white bars). The system threshold, the CUSUM decision interval (solid line), is exceeded on June 30 and remains above threshold until July 9. With relatively low levels of ILI occurring in the summer months, this scenario demonstrates the ability of the system to detect increased ILI visits on weekdays and over the Fourth of July holiday.

**Figure 4:**
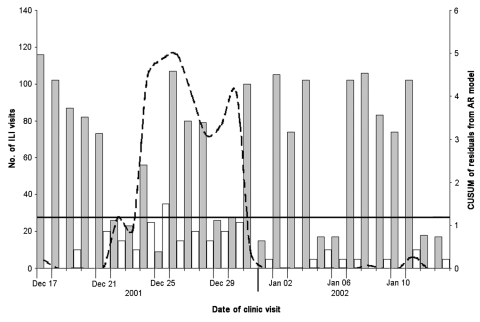
Cumulative sum (CUSUM) control chart of a hypothetical anthrax release occurring December 17, 2001. CUSUM of the residuals (broken line) is charted over the observed number of influenzalike (ILI) visits to the HealthPartners Medical Group (gray bars) and the additional outbreak-associated ILI cases (white bars). The system threshold, the CUSUM decision interval (solid line), is exceeded on December 24 and remains above threshold until December 30. With high levels of ILI occurring in the winter months, this scenario demonstrates the ability of the system to detect increased ILI visits during influenza season and over the winter holidays.

**Figure 5:**
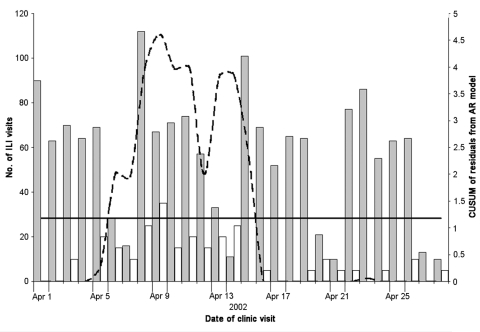
Cumulative sum (CUSUM) control chart of a hypothetical anthrax release occurring April 1, 2002. CUSUM of the residuals (broken line) is charted over the observed number of influenzalike (ILI) visits to the HealthPartners Medical Group (gray bars) and the additional outbreak-associated ILI cases (white bars). The system threshold, the CUSUM decision interval (solid line), is exceeded on April 6 and remains above threshold until April 15. In this scenario, an additional 45 cases of ILI over 5 days are necessary to push the CUSUM above threshold. This represents an 11.7% increase in ILI during that time period.

The hypothetical anthrax release was modeled at three different time periods beginning June 26, 2001, December 17, 2001, and April 1, 2002. The threshold for CUSUM in each scenario was calculated as 1.1812, which resulted in an average-run-length of 50. The outbreak that began in June was detected on June 30, 4 days after the release, with a CUSUM value of 3.09 and after 30 outbreak-associated ILI patients (11.9% increase above expected) visited the HPMG clinic network (Figure 3). The December outbreak was detected 7 days after the release with a CUSUM value of 2.33 and 130 outbreak-associated ILI patients (12.4% increase above expected) (Figure 4). The April outbreak was detected 5 days after the release with a CUSUM value of 2.00 and an additional 45 outbreak-associated patients with ILI (11.7% increase above expected) recorded in the clinic network (Figure 5).

## Discussion

Based on the six criteria we propose, we have attempted to construct a time-series syndromic surveillance system capable of detecting a bioterrorism or other public health event against the background of normal ILI clinic visits. Patient use patterns and seasonality have a considerable effect on the distribution of the dataset, an effect that must be considered when designing the autoregressive model.

Because the HPMG network offers same-day scheduling for its members, many patients do not seek care on the weekend, when only urgent care facilities are open. This delay results in an increased caseload on Monday, a situation that is further exacerbated on a 3-day weekend. The distribution of data is also affected by limited clinic access associated with holidays. The HPMG clinic network operates at a reduced capacity on New Year's Day, Memorial Day, Independence Day, Labor Day, Thanksgiving Day, Christmas Eve Day, and Christmas Day. These holidays often occur on different days of the week from year to year, and therefore generate lower-than-expected counts in the dataset. Additionally, ILI events occur with greater frequency in the winter, which generates a seasonal effect associated with the HPMG ICD-9 data.

Figure 1 shows general agreement between the distribution of ILI in the HPMG clinic network and influenza and pneumonia deaths in the greater metropolitan area during the same period. In the Minneapolis-St. Paul metropolitan area, a lag of 1 to 2 weeks occurs between time of initial signs and symptoms for ILI in HPMG clinics and an increase in influenza and pneumonia related deaths. This lag is less than that noted in other studies (*27*).

Influenza season in Minnesota is variable; onset ranges from early October through mid-January. Figure 2 illustrates a large, sustained increase of ILI beginning December 12, 2000. The Minnesota Department of Health Public Health Laboratory confirmed the season's first positive influenza isolate on December 13, 2000. This signal suggests that the rapid detection of ILI in the community is attainable by monitoring ICD-9 counts representative of ILI in a clinic network. When persons >65 years of age were separated into a distinct ILI syndrome category, a statistically significant signal is observed from November 18 to November 20. This increase in the >65-year category precedes the relatively large signal in the general population by approximately 3 weeks, demonstrating the utility of analyzing subsets of the patients as possible sentinel populations.

The ability of the system to detect additional bioterrorism-related cases is apparent in the hypothetical scenarios illustrated in Figure 3, Figure 4, and Figure 5. When background levels of ILI are relatively low, the system quickly detected additional cases associated with the anthrax release. At best, the system detected the outbreak only 2 days after the first case-patients began to visit the clinics. In winter months, when background ILI is higher, the system was slower to detect the outbreak-associated cases. In December 2001, a 5-day delay occurred between the appearance of symptomatic patients to the clinics and the recognition of the outbreak by the system. Twenty-five additional patients were seen at clinics on December 24, 2001, a holiday, and the system calculated a significant CUSUM alarm of 4.48. The ability of this system to detect the outbreak-associated cases at different times of the year, on weekends, and on holidays shows that the autoregressive model adequately controls for variance and autocorrelation in the dataset.

These scenarios demonstrate that the system possesses the ability to detect the cumulative sum of a small amount of additional counts. The practical success of this surveillance system is limited only by the availability and quality of the source data.

## Conclusion

We have established criteria necessary for initiating syndromic surveillance for ILI and have demonstrated the effectiveness of our detection algorithm by using proxy data for a bioterrorism agent release and historical data for influenza. We believe that this approach to syndromic surveillance is useful in detecting increases in ILI.

## Appendix

We used this SAS code in fitting the autoregressive model (AUTOREG) that generates the residuals used in the cumulative sum analysis (CUSUM).

PROC AUTOREG DATA=WORK._egtemp_;

/* The time-series identifier, dov, is used in any requested plots. */

MODEL sqrt = Holiday dow cos sin /

NLAG=7

MAXITER=50

METHOD=ML

backstep

DW=13 dwprob

;

OUTPUT OUT=SASUSER.Residuals

LCLM=LCLM UCLM=UCLM PM=PREDICTEDM RM=RESIDUALM

R=RESIDUAL ;

RUN;

The dataset is structured to contain a row for each day in the historical file for all influenzalike illness (ILI) visits in the clinic network. In the code, *sqrt* is the variable that contains the square root of the count for all ILI visits on day *dov*. *Holiday* is a dummy-coded variable for regular or nationally observed holidays, and *dow* is a dummy-coded variable for weekday or weekend. *Sin* and *cos* are variables for seasonal adjustments and can be calculated for each *dov* by using the respective formulas, where *x_n_* is a continuously increasing integer: 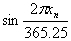
or 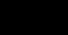.

proc cusum data = SASUSER.residuals;

xchart residual*dov /

maxpanels = 100

interval = date7

mu0 = –0.0027357 /* target mean for process */

sigma0 = 0.7435702 /* known standard deviation */

delta = 2 /* shift to be detected */

h = 1.1812 /* cusum parameter h */

k = 1 /* cusum parameter k */

scheme = onesided /* one-sided decision interval */

tableall

cinfill = ywh

cframe = bigb

cout = salmon

cconnect = salmon

climits = black

coutfill = bilg;

label residual = ´Cusum of Residuals´;

run;

A dataset is created containing the residuals from the autoregression model and used for the one-sided CUSUM analysis. The residuals are charted for each *dov*. The example values of *delta*, *h*, and *k* correspond to an average-run-length (ARL) of 50.
